# Wm. C. Cooper, of Cleves, Ohio, Poet

**Published:** 1896-05

**Authors:** 


### WILLIAM C. COOPER, OF CLEVES, OHIO, POET.

There is but little in the hard task of the physician to lead him into the higher realms of imagination. Placed by the psychologist midway between the intellect and the will, imagination has no place in the stern reasoning demanded in the pursuit of medicine, but on the contrary, it is essential in the attainment of proficiency in the *ars poetica.* Instances are not wholly wanting of souls that have broken the fetters of a rigid and exacting calling, and have soared into the rarified heights of the empyrean. Had Keats been chained to his gallipots, the world would not have been richer for an Endymion, and had not J. G. Holland abandoned the profession of medicine for literature, "Bitter Sweet" would have remained unsung, and had not the divine afflatus found place among the dry bones and processes in the brain of Oliver Wendell Holmes, Darius Green and his unfortunate experience as an æronaut would have been unknown to fame. These are not the only hands that have rolled the pill and swept the poet's star-tuned harp. The news comes from Cleves, Ohio, that another of our co-laborers in the healing art has burst from nebulous obscurity and gives promise of being one of the brightest stars of the constellation.

Let us divest ourselves of all taint of earth and fly with him to the sidereal abode of his heart's Stellaline.

It was love that moved the heart of Wm. C. Cooper, M. D., and when it came with its soft illusions, farewell to the patient toils of science and the labors of the midnight oil. His love had the fatal gift of beauty, that quality of woman by which Adam fell, for was he not drawn to sin by the one gold hair around the slender throat of Lilith, the first love? Who can find it in his heart to censure Dr. Cooper, for he says of his Stellaline (the name is regrettably suggestive of crinoline, vaseline or a German synthetic compound)

"Her beauty is something to dream of and die,
The cast of Camilla's, constraintive and clean,
But intenser and more suggestively high;
'Tis only evoked in the ultramost sky
The beauty of my Stellaline."

No reflection upon Stellaline's morals can possibly be construed from the term suggestively high, for it is expressly stated that she was "constraintive and clean." From such an evolution it is natural to presume that

"She was one with the angels that dwell 'mid the stars,
And they named her, this seraph, for Astraland's queen."

Though of undoubted celestial origin she shows that she was not above a human weakness, an earthly, not to say a barbaric taste for adornment of the person for

"They ravelled out ribbons from rainbow bars
And jewels they caught from the flaming of Mars,
These angels to deck Stellaline."

In the matter of gowns her preference would be likely to arouse the disapproval of young ladies with a proper regard for *les convenances.* The gauzy texture would have displayed her charms in a way to shock the discreet. It was

"Of gossamer sunset and spangled with glints
From the scintillate soul of divine Hippocrene,
Her robe they festooned with empyrean tints
Of Heaven's own jasper and pearl in their tints,
For my soul's sunlight, Stellaline."

The crowning glory of this goodly person, despite the high artificial effects of glints from Hippocrene and tints of gems borrowed from the treasury of the region of the blest, was beyond all odds

"Her mystic, miraculous, marvelous hair
With its gold and its shimmer and glitter and sheen."

Oh, to have been among "they" whose blessed privilege it was to loosen the snood that bound this glimmer and shimmer of gold and through it to

"Sprinkle the sparkle of stars shining there,
And a bouquet of flushes and flashes and flare
They pinned on my own Stellaline."

Not content with dealing out whole bouquets of royal flushes and Fourth of July pyrotechnics, the angelic hosts further show their appreciation of their queen—and Dr. Cooper's—by the bestowal of something more refined than tawdry gewgaws.

"For out in a realm remote from the real
Of Time and its paltry transpirings serene,
They wove for her brow, in this border ideal,
A crown out of blushes and kisses and feal—
All this for my heart's Stellaline."

Allured by the immediate prospect of fairly wallowing in this mire of blushes and kisses and feal (was it feel?), the soul of Dr. Cooper, the poet, soars aloft, leaving behind all mean and paltry transpirings serene, shedding off all professional responsibilities, the diarrhœa unchecked, the placenta undelivered, the vessel still bleeding, the fee unpaid—all earthly considerations are lost to the winds, he must fly at all hazards, and this is the route he selected when he "flew:"

"Through vistas lethean, o'er furthermost pastures
In over-soul transports, in trances serene,
I floated through measureless, visionful vastness,
To her, and absorbed in its ultimate lastness
The presence of sweet Stellaline."

There is a finality about "ultimate lastness" that would preclude the hope of seeing Dr. Cooper again on earth, but should he at any time reappear, having fallen off flaccid and drained from the hermaphrodite embrace of his Stellaline, we bespeak for the privilege of throwing the first brick at him.

